# Chubby, Hairy and Fearless. Subcultural Identities and Predictors of Self-Esteem in a Sample of Polish Members of Bear Community

**DOI:** 10.3390/ijerph17124439

**Published:** 2020-06-20

**Authors:** Magdalena Mijas, Karolina Koziara, Andrzej Galbarczyk, Grazyna Jasienska

**Affiliations:** 1 Department of Environmental Health, Faculty of Health Sciences, Jagiellonian University Medical College, 20 Grzegorzecka St., PL 31531 Krakow, Poland; agalbarczyk@gmail.com (A.G.); jasienska@post.harvard.edu (G.J.); 2 Department of Anthropology, Yale University, 10 Sachem St, New Haven, CT 06511, USA; 3 Institute of Psychology, Faculty of Philosophy, Jagiellonian University, 6 Ingardena St., PL 30060 Krakow, Poland; karolina.koziara@doctoral.uj.edu.pl

**Keywords:** social environment, minority stress, sexual minority, weightism, intersectionality, gay men

## Abstract

Bear subculture exists within a larger gay community, which has been recognized by public health experts as disproportionately burdened with stigma and related health adversities. Bears are distinguished by a particular body look—body hirsuteness and heavy-set physique. Previous research documented the various health risks, and the exposure to both sexual minority and weight stigma, of this population. In this study we focused on the determinants of self-esteem in Bears. We explored the significance of such predictors as: perceived sexual minority and weight stigma, age, resilience, and physique as reflected by the BMI. Our sample consisted of 60 men from the Polish Bear community (i.e., Bears, Cubs, Otters, Wolves). Linear regression models were performed for the entire sample (N = 60) and for Bear-identified men (N = 31). Perceived sexual minority stigma negatively, and resilience positively, predicted self-esteem. In the case of Bear-identified men, age, perceived exposure to weight discrimination, and BMI were also significant predictors of self-esteem. Higher BMI in the case of Bear-identified men predicted higher self-esteem. Our results suggest that although Bear-identified men are characterized by their similarities to other gay men, subcultural identities create unique social contexts that are important for health and health interventions in this population.

## 1. Introduction

Over the last few decades, stigma has been recognized as one of the fundamental drivers of population health [1]. Multiple studies associated exposure to stigma with health disparities in various populations, including the gay and bisexual, and other men who have sex with men (MSM), disproportionately affected by both physical and mental health problems compared to the general population [2,3]. These health inequalities have been explained and studied within the minority stress framework, according to which living in an unfavorable social environment—characterized by prejudice and discrimination towards members of minority groups—is associated with chronically elevated levels of stress, and therefore adversely affects the well-being of burdened populations [4].

Although the health indicators and health-related behaviors of men in sexual minorities have been studied extensively for decades, very few studies have acknowledged the existence of multiple intersecting social identities, and their complex interlocking influence on health in this population [5]. Instead, most previous studies seem to overlook the diversity in sexual minority men, and separately examine various social statuses—such as sexual identity, gender, ethnicity or socioeconomic status—and their associations with health [6]. Only recently have the researchers started to explore the associations between gay men’s health and their subcultural affiliations [7]. The growing body of research has not only demonstrated that various subcultural identities function within this population, but it has also indicated that some of them may have particular relevance to health [8,9].

One such subcultural grouping among gay, bisexual and other sexual minority men is the Bear community. Although the Bear identity and community originated in the US during the 1980s, it quickly became popular worldwide, with central Europe and Poland being no exception. It has been estimated that a Bear identity may be held by about 14–22% of sexual minority men [8,9].

### 1.1. The Bear Subculture

Men who identify as Bears are often characterized as being positioned outside of the mainstream gay community, and as opposing to the stereotypes of gay men as effeminate [10]. They are distinguished by a particular body look—a heavier, muscular or less sculpted physique, which is usually accompanied by more pronounced secondary sex characteristics, such as body hair. As a group, Bears celebrate and eroticize larger bodies, and exhibit resistance to anti-obesity culture, perpetuated by mainstream gay values [11]. Although the individual meaning of Bear identity may, and most certainly does, differ among members of this subculture, it is very common for Bears to affirm traditional notions of masculinity, which signify values of maturity and self-acceptance, and which they reinterpret and redefine to allow for emotional and sexual intimacy with other men [12].

It is worth noting that the Bear community is far from homogenous, and further classification of Bears based on such characteristics as age, physical characteristics or sexual interests has been developed within this subculture [7]. For example, younger men who identify with the Bear community and exhibit typical Bear characteristics, such as being larger and hirsute, are typically referred to as Cubs; older Bears who have grey or silver hair are called Polar Bears; thin and hirsute men are labelled Otters; and men with a lean, muscular physique, who are usually more sexually dominant, identify as Wolves. Finally, men who are attracted to Bears are referred to as Admirers or Chasers [7].

### 1.2. Bear Identity and Health

There is a dearth of research on Bears’ well-being, and most of the existing studies, due to their explanatory and descriptive design, offer rather limited insights into the health and health determinants of this unique population [7]. According to previous studies, men from this community are older [10,13,14], more hirsute [8,15], and are characterized by higher body mass and body mass index, compared to other sexual minority men [8,10,15]. Several studies suggest that this population is burdened with a greater prevalence of high-risk behaviors, such as condomless anal sex with casual partners [14,16], illicit drug use [9] or greater numbers of casual sexual partners [13]. Other studies, however, do not confirm these findings (e.g., [10]).

Perhaps the most comprehensively investigated construct related to health in this population is self-esteem. Several cross-sectional studies compared the levels of self-esteem among Bears with other sexual minority men [8,9,10,15]. Two studies indicated that Bears are characterized by lower self-esteem [8,10], and two others failed to demonstrate any significant difference in self-esteem between Bears and other MSM [9,15]. Lyons and Hosking [10] observed that the difference in self-esteem between Cubs and other sexual minority men was accounted for by the BMI. They interpreted this result as associated with a general cultural preference for thinness. Moskowitz and colleagues [8], who also reported significantly decreased self-esteem among Bears as compared to other MSM, ran an additional analysis to explore whether being a more active member of the Bear community, or deeming this community more important, affected the level of self-esteem, but they found no significant results.

These observations contradict the results of qualitative studies, which suggest that claiming a Bear subcultural identity is perceived by men as a transformatory experience, and leads to a radical change in self-perception, from initial shame and marginalization to feelings of self-acceptance and validation (e.g., [12,17]). The qualitative analyses of Bear experience mostly explored the topic of stigma exposure and management, with a particular focus on weightism (bias or discrimination against people who are overweight), and the strategies adopted by members of this community to challenge body ideals embedded within broader society and mainstream gay culture (e.g., [17,18]).

Bears are portrayed in these studies as struggling with both sexual minority and weight stigmas, which have detrimental effects on their well-being [11]. Although no previous research has quantitatively investigated the associations of weight and sexual minority stigma with self-esteem in Bears, the studies conducted on other groups confirmed this effect (e.g., [19,20]). Obese individuals who experience prejudice and discrimination based on their body weight have been found to report not only decreased self-esteem [19,21], but also increased levels of depression and other negative mental health outcomes [19,22]. Sexual minority stigma has a similarly detrimental effect on mental health, including self-esteem, in sexual minority participants [20,23,24].

The previously mentioned qualitative studies on Bears suggest, however, that at least to some extent the negative impact of prejudice and discrimination on the mental health of Bears is compensated by this subcultural affiliation and identity [11,12,17]. Discovering the Bear community, which offers a space of relief, support and acceptance, challenges the self-perceptions of these men, and eventually results in embracing their physical self and adopting new body ideals [12,17]. Previously stigmatized attributes, such as being hirsute and heavier, may, and often do, become the source of feelings of attractiveness and self-confidence.

This suggests that self-esteem in Bears is influenced by complex, and to some extent contradictory, factors, such as multiple stigma exposure (due to both minority sexual identity and greater body mass) and the empowering self-perceptions associated with claiming Bear identity [12,17,18]. Another factor inherently associated with stigma experience is resilience, which represents the ability to thrive in the face of adversities, and is therefore an essential aspect of any analysis of health determinants in stigmatized populations [25]. Previous studies indicated that resilience buffers the effect of various stressors on health in different populations [26,27].

Without a doubt, more research is needed in order to better understand experiences and health determinants among members of this unique subculture. In this article, we decided to focus on investigating predictors of self-esteem, as this is one of the most comprehensively and consistently explored construct among Bears, and it has been linked in previous studies to both mental and physical health [28].

### 1.3. Theoretical Framework and Hypotheses

This study is grounded in a minority stress framework, which associates experiences of stigma with a unique, chronic and additional psychosocial burden—minority stress [4]. The minority stress model has been proposed as a conceptual framework for explaining the greater prevalence of health adversities among sexual minority populations, as compared to the general population [4,25]. This study is also informed by the concept of intersectionality, which posits that various social identities/statuses are not independent, but instead intersect and interact to create distinct personal realities [29]. According to this perspective, attempting to understand health disparities between various populations via a single analytical category (such as sexual identity or weight status in the case of Bears) ignores the complex ways in which these categories intersect to create health adversities [29]. Instead, the intersectionality perspective proposes the examination of the health of multiply disadvantaged populations in their own contexts, focusing on particular intersections of identities and exploring the interdependence of various identities, rather than summing their distinct effects on health [5].

Based on previous studies, we expect to find in the studied Bear community that: (i) perceived exposure to sexual minority stigma is negatively related to self-esteem; (ii) perceived exposure to weight stigma is negatively related to self-esteem; (iii) greater body mass index (BMI), associated with more desirable body built in this subculture, is positively related to self-esteem; (iv) individual resilience positively predicts self-esteem.

## 2. Materials and Methods

### 2.1. Recruitment and Data Collection

The invitations to participate in the study were distributed among members and supporters of The Bears of Poland Association. We recruited the members of the Bear community through mailing lists, social media, and during various social gatherings addressed to them. Research meetings, held between June and December 2017, included anthropometric measurements and questionnaires. The meetings were held in Krakow and in 3 other major Polish cities during Bear community events. All questionnaires were anonymized (coded with random ID number) to ensure confidentiality, manually checked for completeness, and entered into a database by the first author.

The study was conducted in accordance with the Declaration of Helsinki, and the protocol was approved by the Jagiellonian University Bioethics Committee (122.6120.70.2017). Written informed consent was obtained from the participants.

### 2.2. Participants

We analyzed data obtained from 60 cisgender and gay-identified members of the Polish Bear community. Out of 64 men who participated in the study, 4 persons revealed bisexual identity or stated they do not label their sexual identity. Those men were excluded from further analysis. We decided to include only gay-identified and cisgender men in the analysis, as intersections between various sexual identities (bisexual or gay) and subcultural identities were beyond the scope of this study. The number of participants who labeled their identity as other than gay was also too low to run additional comparisons.

Among the 60 cisgender and gay-identified men aged 25 to 53 years who participated in the study, 31 persons (52%) described their subcultural identity as Bear, 6 men (10%) identified themselves as Chasers or Admirers, 12 men identified themselves as Cubs, Wolves or Otters, and an additional 11 men did not include Bear-affiliation in their identities. As intersections between subcultural identities and health were in the scope of our project, we ran separate models for the whole sample (60 men) and only Bear-identified men (31 men).

### 2.3. Measures

This study included questionnaires and anthropological measurements such as body height and weight. The survey comprised demographic items (e.g., gender identity, age, education, income and place of residence) and such questionnaires as *The Rosenberg Self-Esteem Scale* [30], selected factors of *The Daily Heterosexist Experiences Questionnaire* [31], a modified version of *The Experiences of Discrimination (EOD) Index* [32], and the *Resilience Measurement Scale* [33].

The participants started with answering multiple choice questions about their gender identity (man, woman, transman, transwoman, woman with transgender past, man with transgender past, transgender, transsexual, non-binary, queer, intersex, other) and sexual identity (gay, bisexual, heterosexual, asexual, queer, non-defined, other), which was followed by the question of whether they identify with such categories distinguished within the Bear community as Bear, Cub, Otter or Chaser/Admirer.

We used *The Rosenberg Self-Esteem Scale* (RSES) to assess the self-esteem of the participants. The RSES is a widely used self-reported questionnaire consisting of 10 items to which the participants refer on a 4-point Likert-type scale (from *strongly disagree* to *strongly agree*). The Polish adaptation demonstrates good internal consistency and validity [34]. Higher scores indicate greater self-esteem.

*The Daily Heterosexist Experiences Questionnaire* (DHEQ) was used to assess perceived exposure to stigma connected with minority sexual identity. This questionnaire was developed by Kimberly Balsam and colleagues (2013) to address the issue of heterosexism in the everyday functioning of LGBT community members. It consists of 50 items rated on 6-point Likert-type scale with 0 indicating *did not happen/not applicable to me*; 1 = *it happened, and it bothered me NOT AT ALL*; 2 = *it happened*, *and it bothered me A LITTLE BIT*; 3 *= it happened, and it bothered me MODERATELY*; 4 *= it happened*, *and it bothered me QUITE A BIT*; and 5 *= it happened, and it bothered me EXTREMELY*. The questionnaire includes nine factors, such as: *Victimization*, describing the experience of physical violence on the basis of sexual or gender identity; *Harassment*, capturing the experience of ill-treatment and discrimination; *Family of Origin*, depicting the experience of rejection by family of origin; *Vigilance*, capturing the efforts made to conceal one’s sexual or gender identity; *Isolation*, describing the feelings of loneliness and alienation; *Vicarious Trauma*, depicting the feelings of distress resulting from learning about the discrimination experienced by other members of the LGBT community; *HIV/AIDS Stigma*, capturing the stigma related to HIV; *Gender Expression*, illustrating the experience of ostracism resulting from a non-normative gender expression; and *Parenting*, depicting the stigma experienced by members of LGBT community who are parents. The Polish adaptation is characterized by good psychometric properties [35]. We included seven out of nine DHEQ factors (i.e., *Victimization, Harassment, Family of Origin, Vigilance*, *Isolation*, *HIV stigma* and *Vicarious Trauma*). Higher average scores indicate greater exposure to stigma.

To assess perceived body weight discrimination, we used a modified version of the *Experiences of Discrimination (EOD) Index* used in Year 25 of the Coronary Artery Risk Development in Young Adults (CARDIA) study [36]. The EOD index assesses perceived discrimination, being prevented from doing something, being hassled, or being made to feel inferior because of body weight in 7 different situations, including: at school, at work, at home, getting a job, housing, medical care and in a public setting/in the street. Having consulted gay men, we added one more item to capture their weight stigma experiences: “on the Internet, while using social media and networking apps, chats, or dating web sites”. The overall index scores ranged therefore from 0 to 8.

*The Resilience Measurement Scale SPP-25* [33] used in this study is a Polish questionnaire aimed at capturing resilience understood as a personality-like trait reflecting personal ability to deal with both traumatic and everyday stressors. The questionnaire consists of 25 statements rated on a 5-point Likert-type scale with regard to how well each statement describes the participant (with 0 meaning *definitely not,* and 4 meaning *definitely yes*). Higher average score indicates greater resilience to stress.

Body height was measured with a stadiometer in the standardized position. Body weight was measured with a Tanita BC-545 Segmental Body Composition Monitor. Waist circumference was measured with stretch-resistant measuring tape at the midpoint between the lower margin of the last palpable rib and the top of the iliac crest, according to World Health Organization STEPwise Approach to Surveillance (WHO STEPS) protocol [37].

### 2.4. Data Analysis

Data organizing and statistical analyses were performed by the means of R Studio [38]. Since our study sample consisted of both Bear-identified men and other men from the Bear community (e.g., Wolves, Otters, Cubs, as well as men who self-reported community affiliation but did not include that aspect in their identities), and since one of the aims of our study was to examine how sexual minority and weight stigma intersect in the context of subcultural identities, we created a subset of data for the Bear-identified men only. All analyses were therefore performed for the whole study sample (further referred to as ‘All sample’, N = 60) and for the Bear-identified men only (further referred to as ‘Bear-identified men’, N = 31).

In order to make the interpretation of the data more comprehensive, all predictors included in the model were grand mean centered. The estimations were computed using regular linear model with all predictors included at once. We also employed the QuantPsyc package [39] and Companion to Applied Regression (CAR) package [40] to estimate Betas and variance inflation factors (VIF).

Two separate linear regression models for both of the distinguished groups (‘All sample’ and ‘Bear-identified men’) were calculated with self-esteem (RSES) as a dependent variable, and age, the BMI, perceived sexual minority stigma (DHEQ), perceived weight stigma (EOD) and resilience (SPP-25) as predictors. The VIF and Tolerance values met the required criteria for both models to be approved.

## 3. Results

The demographic data with respect to the distinguished groups of participants are presented in Table 1. The majority of study participants lived in some of the largest Polish cities, and had at least some history of university education. Approximately one fifth of the sample reported having some financial hardships (monthly income either hardly sufficient or insufficient to cover basic needs). Of all studied men, 8% were characterized by a BMI within the normal range, 30% had a BMI indicative of overweight, and 62% were obese. In the case of Bear-identified men, 16% had a BMI indicative of overweight, and 84% of participants were obese. The BMIs for the whole sample ranged from 21.3 to 49.1 kg/m^2^.

In the Bear-identified sample, all of the predictors were statistically significant (Table 2). Age, perceived exposure to sexual minority stigma and weight stigma negatively predicted self-esteem, and both BMI and resilience positively predicted the self-esteem of Bear-identified men.

When the same model was applied for the entire sample, only exposure to sexual minority stigma and resilience remained significant predictors of self-esteem (Table 3). In the case of all participants, perceived exposure to sexual minority stigma negatively predicted self-esteem, while resilience predicted it positively.

## 4. Discussion

In this study we quantitatively explored the predictors of self-esteem in a group of Polish gay-identified men who identify with Bear subculture. Our analysis was grounded in both minority stress [4] and intersectionality frameworks [29], and highlighted the extent to which Bear-identified gay men are characterized by shared and unique predictors of self-esteem, as compared to other members of the Bear community. Although to some extent all the study participants share common experiences, which is reflected by associations between perceived sexual minority stigma and self-esteem, claiming Bear identity seems to be associated with unique psychological dynamics.

Consistent with minority stress theory [4], perceived exposure to sexual minority stigma and the resilience level were found to be significantly related to self-esteem in both groups. Greater exposure to sexual minority stigma was connected with lower self-esteem, while greater resilience was associated with higher self-esteem. In the case of Bear-identified men, however, age, perceived exposure to weight discrimination and BMI were also significant predictors of self-esteem.

Among Bear-identified men, both perceived exposure to sexual minority stigma and body weight stigma negatively predicted the levels of self-esteem. This result is consistent with previous qualitative analyses, which indicated that Bears in the social environment are exposed to both types of stigma, and both sexual minority, and weight prejudice and discrimination, affect their well-being [17,18]. Although there is limited data on the prevalence of weight stigma in Poland, discrimination and prejudice against sexual minority persons remain widespread [41]. Poland has only recently been rated by ILGA-Europe as the country with the worst legal and human rights situation for LGBT people among EU member states [42]. This calls for greater involvement of public health experts in raising awareness among policy makers about the negative impact of discrimination and stigma on health. Our observations are also in line with the previous studies, which demonstrated associations between stigma exposure and negative mental health outcomes in obese as well as sexual minority individuals [19,20,23,24]. Therefore, this study adds to the growing literature on the health implications of stigma and related stress.

Both exposure to sexual minority and weight stigma explain the significant amount of variance (Table 2) in the self-esteem of Bear-identified men in our study. Given the previous findings, which indicated that adults with multiple disadvantages are more likely to experience health adversities such as major depression, poor physical health, and functional limitations [43], this alarming result demands that special efforts be made by public health experts to reach and support this unique community.

Another factor that negatively predicted self-esteem in a subgroup of Bear-identified men was age. This result contradicts both the previous studies on sexual minority men and the studies on the general population [44,45]. According to a recent meta-analysis of longitudinal research on the development of self-esteem within lifespan, self-esteem increases strongly in young adulthood, continues to increase in middle adulthood, and peaks between the ages of 60 and 70 years. This pattern is consistent across such variables as gender, country and or birth cohort [45]. The studies on sexual minority men similarly indicated that gay men in midlife, compared to younger sexual minority adults, are characterized by better mental health and higher self-esteem [44,46,47]. It is possible that Bear-identified men experience a decrease in self-esteem with age due to, for instance, health issues, or as a result of cumulative exposure to multiple stigmatizations. It is also possible that other factors included in the regression model explain most of the variance in self-esteem which is usually associated with age. Future studies should explore the associations between self-esteem and age among members of this subculture, preferably from a longitudinal perspective.

Consistent with previous studies on resilience and its associations with health-related constructs, the present study found that among both distinguished subsamples (all participants and the Bear-identified ones), resilience positively predicted the levels of self-esteem [25]. Resilience has been recognized by the minority stress model as an essential component of stress experience, and defined as a process of stress buffering [4,25]. It therefore constitutes a promising site for health promotion interventions in this and other stigmatized communities.

Supporting previous qualitative research [12,17], our quantitative analysis also indicated that among Bear-identified men, the body mass index positively predicted self-esteem, when controlling for age, stigma exposure and resilience in a regression model. Higher BMI was associated with increased self-esteem only among men who identified as Bears, which suggests that unique processes associated with claiming Bear identity may be responsible for this effect, and most likely distinguish Bear-identified men from other sexual minority men. Previous qualitative analyses indicated that not only does the Bear community offer a site for resistance to the dominant weight stigma and body ideals perpetuated by mainstream gay culture, but it also inspires a radical change in self-perception [18].

This result also supports the recommendations proposed by the intersectionality theory and the framework for research on the associations between stigma and health [5,29]. We incorporated these recommendations by exploring the intersection of sexuality and weight status in a specific context of subcultural identities, exploring the diversity within the studied population, and examining separately the experiences of Bear-identified men and all members of the Bear community, rather than using their subcultural identities as predictor variables. This analytic strategy resulted in novel insights into the subcultural determinants of self-esteem among gay men.

Adopting Bear identity was characterized in previous studies as a turning point, a second coming out, which facilitates self-acceptance and the internalization of new body size ideals [17]. It is possible that the non-Bear-identified participants in our study hold somewhat different body ideals, which is supported by their subcultural identities as Wolves, Cubs and Otters, or by resignation from identifying with any of the distinguished categories. This claim is supported by at least one previous study, which indicated that the difference in self-esteem between Cubs and sexual minority men with no subcultural affiliation was accounted for by the difference in the BMI between those groups [10]. Unfortunately, the number of participants identifying as Cubs, Otters, Wolves, or those who refrained from adopting Bear identity in our sample was too small to run separate models. Future studies should explore those differences both quantitatively and qualitatively. Although in the case of Bear-identified men from our sample the BMI ranged from 25.1 to 48.1 kg/m^2^, the largest proportion of this subsample was characterized by a BMI between 30 and 35 kg/m^2^. It is also possible that the positive association between the BMI and self-esteem is limited to this particular range of the BMI.

This study has some limitations, including the relatively small sample size, the cross-sectional design, and the fact that its results are limited to gay-identified men only. Although it is very likely that subcultural identities, such as the Bear identity, are adopted by other sexual minority men, their number in our sample was too small (N = 4) to run additional comparisons. Our sample also consisted of men who were willing to have their body measurements taken, which suggests that this study design could somehow privilege men with positive associations between body mass and self-esteem. It is therefore possible that our sample consisted of men who felt particularly confident and comfortable with their body size—they did not mind having their body weight and circumferences measured. Future studies should explore these associations, using both body measurements and self-reported data on weight and height, as well as various sampling methods, such as Respondent Driven Sampling, which compensates for non-random sampling in hard-to-reach populations [48].

Nonetheless, these limitations should not overshadow the advantages of this study. First, our project integrated both the minority stress and intersectionality frameworks, in order to investigate how various stigmatized statuses intersect to create health disparities in the context of the subcultural affiliations of gay men. Given that there is a dearth of research examining the intersectional minority stress processes and associations between subcultural affiliations and health in gay men, this study provides a significant contribution to the literature [5]. This study is also the first to examine health-related constructs and their predictors among members of the Polish Bear community. Most of the previous studies among Bears have been conducted in the US, Australia or Canada, and comparable analyses on other samples are lacking. Our study addresses this gap in the literature.

Our models also explained the relatively large degree of variance in self-esteem (54–75%) in the studied sample, especially in the case of Bear-identified men. This suggests that we managed to capture the most important predictors of self-esteem in this unique population. Given the associations of self-esteem with depression [49,50], anxiety [50,51], substance use and other health-related behaviors, such as smoking [52,53,54,55], as well as self-rated health [56,57] and general well-being [58,59], this finding has more far-reaching implications for both mental and physical health.

## 5. Conclusions

In this study, we demonstrated that although Bear-identified men are, to some extent, characterized by similarities to other gay men, the subcultural identities among gay men create unique social contexts important for both their health and health interventions. Within these subcultural contexts, the meaning of various characteristics (including socially disadvantaged ones, such as overweight or obesity) becomes reinterpreted, and may even be reversed.

These results have potential practical implications for health promotion interventions addressed to the members of the Bear subculture, and suggest that links between the subcultural identities of sexual minority men and their health deserve more attention. Due to weight and obesity stigma prevalent in public health campaigns, members of this community may be particularly resistant to messages concerning physical health [18]. This in turn suggests that alternative, weight-inclusive paradigms for thinking about health, which focus on healthier lifestyles regardless of body size, may be more effective for the prevention of chronic disease in this population [60]. The health promotion interventions addressed to this unique community should therefore focus more on encouraging healthy behaviors, such as physical activity, than on increasing the body preoccupation and weight stigma associated with the pressure to lose weight. Furthermore, given their disproportionate exposure to discrimination and prejudice, Bear-identified men may face additional disadvantages in health compared to other sexual minority men [43]. This complexity needs to be taken into consideration by the public health experts when addressing the needs of this community in health promotion initiatives [18]. Tailoring health promotion messages addressed to gay men around their subcultural identities, and taking special effort to reach those communities that face multiple stigmas, may further strengthen those interventions, and contribute to reducing the barriers to adopting healthier lifestyles in the MSM population [10].

## Figures and Tables

**Table 1:** The characteristics of all participants (N = 60) and of Bear-identified men (N = 31).

	All Sample	Bear-Identified Men
Age	35.8 (7.4)	37.8 (7.5)
Education—proportion of university education	0.68	0.67
Place of residence—proportion of > 500,000 inhabitants	0.56	0.61
Income—proportion of insufficient income	0.18	0.19
BMI	32.3 (6.3)	34.7 (6.0)
Waist circumference (cm)	110.5 (16.9)	116.7 (16.5)
Perceived sexual minority stigma	1.3 (0.61)	1.4 (0.7)
Perceived weight discrimination	2.3 (1.8)	2.5 (1.9)
Resilience	3.7 (0.6)	4.04 (0.6) *
Self-esteem	30.7 (5.6)	32.1 (5.3)

* median (IQR).

**Table 2:** The predictors of self-esteem in the group of Bear-identified men (N = 31) tested by linear regression model.

	Estimates (95% CI)	SE	Beta	*p*	VIF	Tolerance
Intercept	32.10 (31.10, 33.09)	0.48	-	**<0.001**	-	-
Age	−0.32 (−0.48, −0.16)	0.08	−0.45	**0.036**	1.41	0.71
BMI	0.45 (0.23, 0.67)	0.11	0.51	**<0.001**	1.70	0.59
Sexual minority stigma (DHEQ)	−2.86 (−4.48, −1.24)	0.79	−0.38	**0.001**	1.27	0.79
Weight stigma (EOD)	−0.76 (−1.43, −0.09)	0.33	−0.27	**0.029**	1.63	0.61
Resilience (SPP-25)	5.54 (3.51, 7.58)	0.99	0.55	**<0.001**	1.11	0.90
F (*DFs*)	18.53 (5, 25)					
Adjusted *R^2^*	0.75					
Overall model’s *p*	<0.001					

**Table 3:** The predictors of self-esteem for All sample (N = 60) tested by linear regression model.

	Estimates (95% CI)	SE	Beta	*p*	VIF	Tolerance
Intercept	30.70 (29.71, 31.70)	0.50	-	**<0.001**	-	-
Age	−0.09 (−0.23, 0.06)	0.07	−0.11	0.254	1.19	0.85
BMI	0.14 (−0.07, 0.34)	0.10	0.15	0.186	1.65	0.61
Sexual minority stigma (DHEQ)	−2.07 (−0.74, 0.58)	0.86	−0.22	**0.019**	1.09	0.91
Weight stigma (EOD)	−0.08 (−0.74, 0.58)	0.33	−0.03	0.804	1.46	0.68
Resilience (SPP-25)	6.77 (5.04, 8.51)	0.87	0.70	**<0.001**	1.01	0.99
F (*DFs*)	14.62 (5, 54)					
Adjusted *R^2^*	0.54					
Overall model’s *p*	<0.001					
